# The Effect of Prophylactic Dexmedetomidine on Hemodynamic Disturbances to Double-Lumen Endotracheal Intubation: A Prospective, Randomized, Double-Blind, and Placebo-Controlled Trial

**DOI:** 10.1155/2013/236089

**Published:** 2013-07-29

**Authors:** Tanyong Pipanmekaporn, Yodying Punjasawadwong, Somrat Charuluxananan, Worawut Lapisatepun, Pavena Bunburaphong

**Affiliations:** ^1^Clinical Epidemiology Program, Faculty of Medicine, Chiang Mai University, Chiang Mai 50200, Thailand; ^2^Department of Anesthesiology, Faculty of Medicine, Chiang Mai University, Chiang Mai 50200, Thailand; ^3^Department of Anesthesiology, Faculty of Medicine, Chulalongkorn University, Bangkok 10330, Thailand

## Abstract

The purpose of this study was to determine the effect of dexmedetomidine on hemodynamic responses to DLT intubation compared to placebo and to assess the adverse effects related to dexmedetomidine. Sixty patients were randomly allocated to receive 0.7 **μ**g/kg dexmedetomidine (*n* = 30) or normal saline (*n* = 30) 10 minutes before general anesthesia. Systolic blood pressure (SBP), diastolic blood pressure (DBP), mean arterial pressure (MAP), heart rate (HR), and rate pressure product (RPP) between groups were recorded. During intubation and 10 minutes afterward (T1-T10), the mean SBP, DBP, MAP, HR, and RPP in the control group were significantly higher than those in the dexmedetomidine group throughout the study period except at T1. The mean differences of SBP, DBP, MAP, HR, and RPP were significantly higher in the control group, with the value of 15.2 mmHg, 10.5 mmHg, 14 mmHg, 10.5 beats per minute, and 2,462.8 mmHg min^−1^. Four patients in the dexmedetomidine group and 1 patient in the control group developed hypotension, while 2 patients in the dexmedetomidine group had bradycardia. Prophylactic dexmedetomidine can attenuate the hemodynamic responses to laryngoscopy and DLT intubation with minimal adverse effects. This trial is registered with ClinicalTrials.gov NCT01289769.

## 1. Introduction

A double-lumen endotracheal tube (DLT) is a device frequently used in thoracic surgery. It effectively provides lung separation and facilitates changing from two- to one-lung ventilation [1]. A previous study found that both laryngoscopy and DLT intubation could stimulate significant hemodynamic responses and increase the plasma concentration of catecholamines [2]. Although these hemodynamic responses including tachycardia and hypertension are transient, they can be harmful to patients with hypertension, myocardial ischemia, or cerebrovascular disease [3, 4]. The supplement of an inhaled anesthetic agent during anesthetic induction and intubation using a nondepolarizing muscle relaxant is a normal practice at our hospital. However, we observe that this practice is found ineffective to attenuate the hemodynamic responses to intubation and rescue treatment is frequently required. Therefore, some measures should be added in order to optimize this condition. 

Several drugs have been used to attenuate the hemodynamic responses to laryngoscopy and DLT intubation. Dexmedetomidine, a highly selective *α*2 receptor agonist, produces sedative, analgesic, and central sympatholytic effects. It reduces norepinephrine release at the neuroeffector junction, inhibits neurotransmission of sympathetic nerves, and decreases plasma catecholamines, which results in a slight decrease in blood pressure and lowering of the heart rate [5, 6]. On the other hand, dexmedetomidine can also increase SBP, the effect mediated by peripheral *α*2 receptor agonism at the vascular smooth muscle. This effect is associated with a higher dose of dexmedetomidine or rapid drug administration [7]. Dexmedetomidine has been used to attenuate the hemodynamic responses to single-lumen endotracheal tube intubation [8–12]; however, its effect on the hemodynamic responses during DLT intubation has not been investigated. The purpose of the present study is to determine the effect of dexmedetomidine on the hemodynamic responses to DLT intubation compared to placebo and to assess the adverse effects related to dexmedetomidine.

## 2. Methods

**Figure 1:**
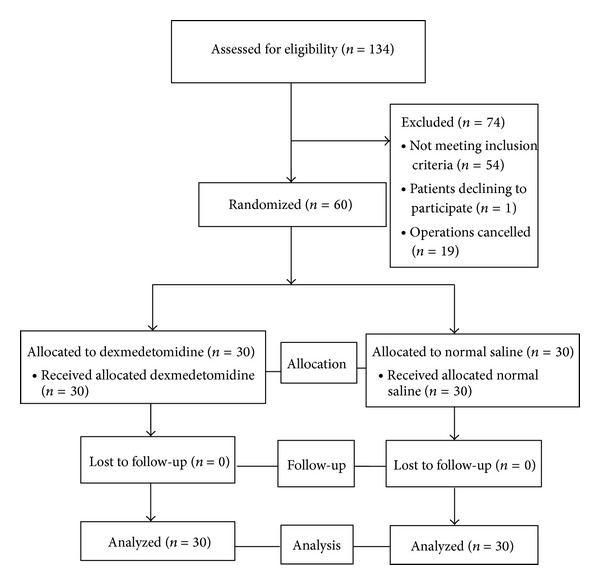
The Consolidated Standards of Reporting Trials flow diagram of the study.

 After approval from the institutional review board and written informed consent were obtained, 60 patients with the American Society of Anesthesiologists (ASA) physical status I-III, aged 18–65 years, undergoing scheduled thoracic surgery between March and December 2011 were enrolled in the study. Exclusion criteria were preoperative arrhythmias, bradycardia (heart rate < 50 beats per minute), second- or third-degree atrioventricular block, tachycardia (heart rate > 100 beats per minute), coronary artery disease, previous cerebrovascular accident, poorly controlled hypertension, renal or liver impairment and suspected difficult intubation. All patients had 0.2 mg/kg of diazepam orally two hours before surgery. In the operating theater the following monitoring was established: noninvasive blood pressure monitoring, electrocardiography (ECG) using the three-lead electrode system, peripheral oxygen saturation, and radial artery catheterization with a 20 G catheter. Patients were randomly allocated into two groups: the dexmedetomidine group and the control group, using a block of four randomizations with each randomized number concealed in a sealed, opaque envelope which would be opened after radial artery catheterization. The dexmedetomidine group received dexmedetomidine, 0.7 *μ*g/kg in 0.9% NaCl 20 mL, while the control group received 0.9% NaCl 20 mL. Both the dexmedetomidine solution and 0.9% NaCl were prepared by a nurse who was not involved in the study so that the investigators would be unaware of the group identity. The designated solution was infused over 10 minutes before the induction of anesthesia. Propofol 2.5 mg/kg and fentanyl 1 *μ*g/kg were given followed by rocuronium 0.6 mg/kg to facilitate endotracheal intubation. Anesthesia was maintained with 1% vaporizer setting of concentration of sevoflurane until tracheal intubation. Four minutes later, the laryngoscopy and endotracheal intubation were performed using a 39 Fr, 37 Fr, or 35 Fr endobronchial tube as recommended by Slinger and Campos [13]. All intubation attempts were performed by the same anesthesiologist (TP). Hemodynamic parameters (systolic blood pressure (SBP), diastolic blood pressure (DBP), mean arterial pressure (MAP), heart rate (HR), and rate pressure product (RPP, SBP × HR)) were recorded at baseline, just before intubation, during intubation, and every minute after intubation for 10 minutes. Recording of parameters including intubation time (time from applying a laryngoscopy to tracheal cuff inflation) was done by another nurse who was not involved in the study. The incidences of hypotension (a decrease in SBP > 25% of baseline or SBP < 90 mmHg) and bradycardia (a decrease in HR > 25% of baseline or HR < 50 beats per minute) were also noted. Rescue medication was comprised of ephedrine 3 mg increments for hypotension (SBP < 90 mmHg for 60 seconds), nicardipine 0.5 mg increments for hypertension (SBP > 200 mmHg or DBP > 120 mmHg for 60 seconds) [2, 14, 15], and atropine 0.3 mg increments for bradycardia (HR < 50 beats per minute). The flow diagram of the study, recommended by the Consolidated Standards of Reporting Trials, is presented in Figure 1.

The primary endpoint of this study was to determine the differences of hemodynamic parameters between groups. The secondary endpoint was the adverse effects of dexmedetomidine such as hypotension, hypertension, bradycardia, or airway obstruction during study drug administration. Based on our pilot study, a sample size of 24 patients per group was required to detect a 25% change of MAP between groups with a power of 90% at the 5% significant level. However, we decided to recruit 30 patients per group to compensate for 20% of dropouts. All statistical analysis was performed using Stata software (version 11.0, College station, TX, USA). Data were presented as mean ± SD for continuous data and number and percent for categorical data. The Shapiro-Wilk test was used to determine normal distribution of data. Statistical analyses were performed using Fisher's exact probability test, unpaired *t*-test, or the Mann-Whitney *U* tests as appropriate. If the difference of the hemodynamic parameters between groups is marginally insignificant, a multi-level mixed model would be analyzed. The multilevel mixed model was used to investigate the effects of group, time, and individuals. The number needed to treat (NNT) was calculated to demonstrate the efficacy of dexmedetomidine administration and defined as numbers of patients needed to be treated to prevent one episode of an increase in MAP more than 25% after intubation compared to baseline value. A *P* value lower than 0.05 was considered to be statistically significant. Statistical analysis was based on the intention to treat.

## 3. Results

**Figure 2:**
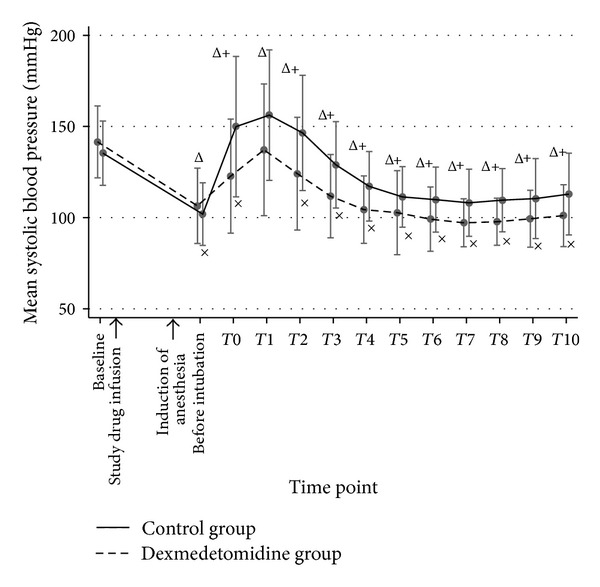
A line graph demonstrated the mean SBP and their standard deviation comparing groups at different time points. Baseline refers to the point of time before the study drug is administered. Before intubation refers the point of time after anesthetic induction just before DLT intubation. T0 is the period during intubation. T1–T10 refer the points of time as minutes after intubation. SBP: systolic blood pressure. ^+^ *P* value < 0.05 compared between groups. ^Δ^ *P* value < 0.05, significant difference compared with baseline in the control group. ^×^ *P* value < 0.05, significant difference compared with baseline in the dexmedetomidine group.

**Figure 3:**
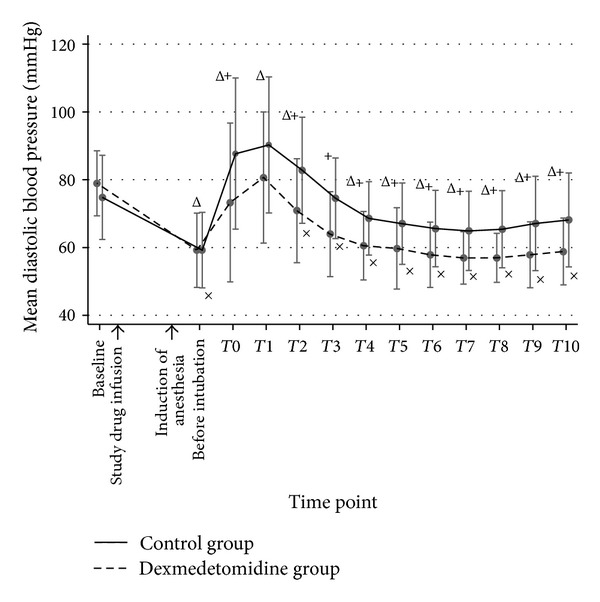
A line graph demonstrated the mean DBP and their standard deviation comparing groups at different time points. Baseline refers to the point of time before the study drug is administered. Before intubation refers the point of time after anesthetic induction just before DLT intubation. T0 is the period during intubation. T1–T10 refer the points of time as minutes after intubation. DBP: diastolic blood pressure. ^+^ *P* value < 0.05 compared between groups. ^Δ^ *P* value < 0.05, significant difference compared with baseline in the control group. ^×^ *P* value < 0.05, significant difference compared with baseline in the dexmedetomidine group.

**Figure 4:**
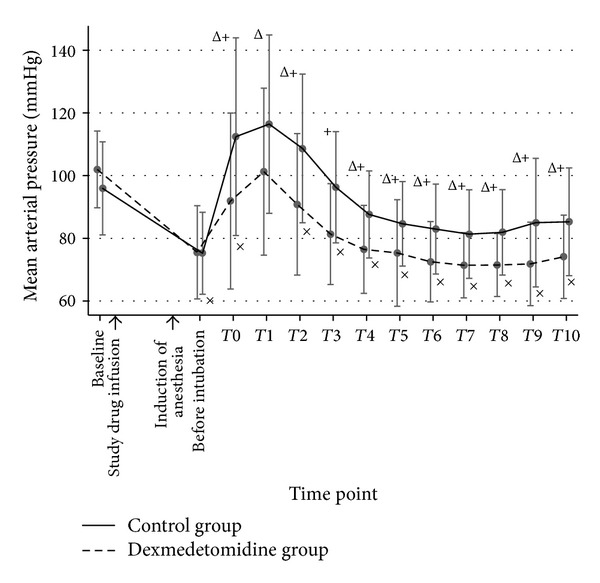
A line graph demonstrated the mean MAP and their standard deviation comparing groups at different time points. Baseline refers to the point of time before the study drug is administered. Before intubation refers the point of time after anesthetic induction just before DLT intubation. T0 is the period during intubation. T1–T10 refer the points of time as minutes after intubation. MAP: mean arterial pressure. ^+^ *P* value < 0.05 compared between groups. ^Δ^ *P* value < 0.05, significant difference compared with baseline in the control group. ^×^ *P* value < 0.05, significant difference compared with baseline in the dexmedetomidine group.

**Figure 5:**
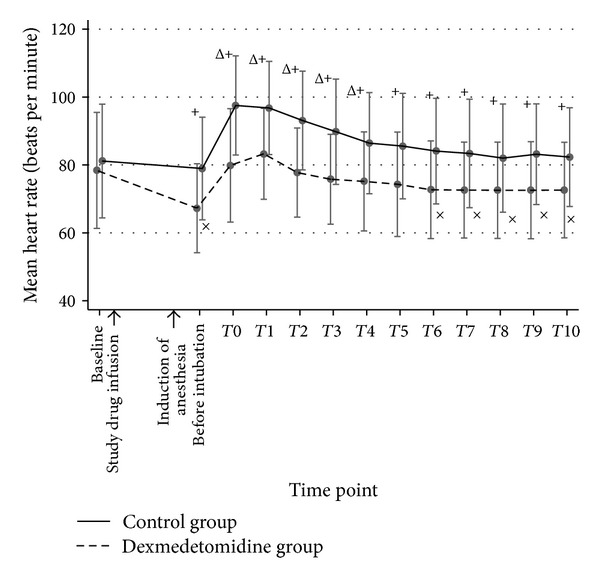
A line graph demonstrated the mean HR and their standard deviation comparing groups at different time points. Baseline refers to the point of time before the study drug is administered. Before intubation refers the point of time after anesthetic induction just before DLT intubation. T0 is the period during intubation. T1–T10 refer the points of time as minutes after intubation. HR: heart rate. ^+^ *P* value < 0.05 compared between groups. ^Δ^ *P* value < 0.05, significant difference compared with baseline in the control group. ^×^ *P* value < 0.05, significant difference compared with baseline in the dexmedetomidine group.

 Sixty patients were randomized and completed the study. Patient characteristics, baseline hemodynamic variables, and intubation time were comparable between both groups (Table 1). Before intubation, mean SBP, DBP, MAP, and RPP in both groups were not significantly different; however, mean HR in the dexmedetomidine group was significantly lower than that of the control group (*P* < 0.05). During DLT intubation (T0) and 10 minutes afterward (T1–T10), the mean SBP, DBP, MAP, HR, and RPP in the control group were significantly higher than those of the dexmedetomidine group throughout the study period except T1, in which the blood pressures (BP) were not statistically different (Figures 2, 3, 4, and 5). A maximal increase of mean HR was observed at T0 in the control group, while a maximal increase of the mean HR in the dexmedetomidine group occurred at T1. Meanwhile, the mean differences of SBP, DBP, MAP, HR, and RPP were significantly higher in the control group between baseline, before intubation, during intubation, and T1–T10 after being adjusted for age, gender, ASA, concurrent disease, intubation time, baseline hemodynamic variables, and intubation attempts (*P* < 0.001) as shown in Table 2. In the dexmedetomidine group, the mean SBP, DBP, and MAP decreased significantly from the baseline before intubation, during intubation, and all through 10 minutes afterward except at T1 (*P* < 0.05). The mean SBP before intubation was 106.5 ± 20.7 mmHg, while the lowest mean SBP was observed at T7 (97.2 ± 13.2 mmHg). Significant decreases of mean HR from the baseline were before intubation and from T6 to T10, with the lowest mean value of 67.3 ± 13.1 beats per minute before intubation (*P* < 0.05). The mean values of SBP, DBP, MAP, HR, and RPP at different time points in both groups were presented in Table 3.  Table 4 shows the incidences of adverse effects during and after intubation, which were not significantly different between groups. Four patients in the dexmedetomidine group and 1 patient in the control group developed hypotension, all of whom but 1 in the dexmedetomidine group required rescue medications. No patient in the control group had bradycardia. In the dexmedetomidine group, 2 patients had bradycardia and only 1 patient required treatment. All patients who received rescue medications responded very well to treatment. There was no episode of respiratory obstruction observed during administration of the study drug. NNT to prevent an increase of MAP more than 25% of baseline for dexmedetomidine was 10 (95% CI: 3.4–10.6). All patients were able to be extubated after the operation. 

## 4. Discussion 

An inhaled anesthetic agent is commonly used in combination with an intravenous anesthetic agent before a nondepolarizing muscle relaxant reaches its onset. In our practice, 1% concentration of sevoflurane may be sufficient to prevent awareness during induction of anesthesia [16]; however, this concentration does not effectively attenuate the hemodynamic responses to intubation. This study found that the mean SBP, DBP, MAP, HR, and RPP in the control group were significantly higher than those of the dexmedetomidine group all through 10 minutes after DLT intubation, except mean SBP, DBP, and MAP between groups at T1. Corresponding to a previous study, this study showed that maximal increases of blood pressure and heart rate occurred 1 to 2 minutes after DLT intubation in the control group and returned to baseline within 5 minutes [2]. Thompson et al. showed that an increase of hemodynamic responses after DLT intubation in patients with an adequate depth of anesthesia was associated with an increase of the plasma level of norepinephrine [2]. In addition, previous studies reported that RPP was one of the major determinants of myocardial oxygen consumption and RPP > 20,000 mmHg min^−1^ could precipitate angina pectoris [17]. Four patients (13%) in the control group and 1 patient (3%) in the dexmedetomidine group had an RPP of more than 20,000 mmHg min^−1^, which could increase the risk of myocardial ischemia [17, 18]. The effect of dexmedetomidine on lowering SBP, HR, and RPP could decrease the myocardial oxygen requirement and may be advantageous for patients at risk of coronary artery disease [19]. Moreover, the effect of dexmedetomidine in the controlling of blood pressure and heart rate during intubation may be beneficial in patients with preexisting hypertension or risk of stroke [20].

Various dosages of preoperative dexmedetomidine ranging from 0.5 to 2 *μ*g/kg have been investigated to attenuate the sympathoadrenal and hemodynamic responses following single-lumen endotracheal tube intubation [8, 10, 12, 21]. A randomized controlled trial study demonstrated that the use of 0.5 *μ*g/kg of dexmedetomidine could decrease the HR and MAP at the 10th minute after intubation compared to placebo in patients undergoing general surgery [22]. However, the HR seemed to be inadequately attenuated few minutes after intubation as observed from the result of the former study. Moreover, the investigators did not compare hemodynamic parameters between groups at that time interval in their study. The use of 1 *μ*g/kg of dexmedetomidine in patients undergoing general surgery could lower both HR and MAP more effectively than the use of fentanyl alone 1, 3, and 5 minutes after intubation and reduce the requirement of intraoperative fentanyl and isoflurane [12]. The administration of 2 *μ*g/kg of dexmedetomidine lowered SBP, DBP, and HR significantly compared to a placebo and reduced the intraoperative isoflurane requirement [21]. During our pilot study, the investigator began with 1 *μ*g/kg of dexmedetomidine infusion over 10 minutes and we found that some patients developed severe hypotension and bradycardia after the induction of general anesthesia and intubation. Therefore, we decided to decrease dose of dexmedetomidine to 0.7 *μ*g/kg in order to minimize its adverse effects.

In the dexmedetomidine group, mean HR and RPP were significantly lower than those in the control group all through 10 minutes after intubation. Maximal increases of blood pressures in the dexmedetomidine group occurred at T1 and they were not significantly different from those in the control group. However, these differences were marginally insignificant. In addition, we observed that some patients had exaggerated hemodynamic responses during and after intubation. The analysis of average mean difference between groups at different time points is may be inappropriate in this situation. Therefore, the multilevel mixed model analysis was then used to evaluate the differences of hemodynamic parameters between groups throughout the study period. The results showed that mean differences adjusted for multiple variables of SBP, DBP, and MAP were significantly higher in the control group than those of the dexmedetomidine group over 10 minutes after intubation. However, dexmedetomidine at dose of more than 0.7 *μ*g/kg may be required in order to ensure the complete abolishment of hemodynamic responses at T1. 

Hypotension and bradycardia are common adverse hemodynamic effects in patients receiving dexmedetomidine as a result of decreasing the central sympathetic nervous activity or preserving the baroreceptor reflex [7]. In the present study, only a few patients had significant hypotension and bradycardia during or after dexmedetomidine administration and responded well to rescue medications. Hypotension and bradycardia occur frequently in patients receiving an initial loading dose of dexmedetomidine especially with rapid administration and also in those with concurrent negative chronotropic medications [19]. Previous studies reported that the undesirable effects of dexmedetomidine were dose related. Basar et al. reported that the incidence of bradycardia after a single dose of 0.5 *μ*g/kg of dexmedetomidine was about 5% [8], while a study of Sulaiman et al. showed development of hypotension or bradycardia with a similar dosage of dexmedetomidine [22]. The incidence of hypotension occurred by about 20% with the use of 1 *μ*g/kg of dexmedetomidine in patients undergoing coronary artery bypass graft surgery [11]. Nevertheless, transient tachycardia, hypertension, and oxygen desaturation were observed after the use of 1 *μ*g/kg of dexmedetomidine in the study of Bajwa et al. [12]. A randomized controlled trial investigated the effect of 2 *μ*g/kg of dexmedetomidine and placebo on anesthetic requirement and perioperative hemodynamic stability. The former study reported that the incidences of intraoperative hypotension, bradycardia, and partial airway obstruction were 20%, 24%, and 12%, respectively. In addition, 56% of patients in the dexmedetomidine group developed bradycardia in the postanesthesia care unit and subsequently had a significantly longer stay in the postanesthesia care unit compared to the placebo group [21]. In this study, although we did not observe any adverse event during the use of 0.7 *μ*g/kg of dexmedetomidine in the pilot study, hypotension (13%) and bradycardia (7%) were observed after intubation in the main study. However, we could not detect any difference in adverse effects between groups. 

 There were a few limitations of this study. First, we did not measure the plasma concentration of catecholamines. Therefore, we could not demonstrate the effectiveness of this dosage of dexmedetomidine in decreasing the sympathetic nervous system activity. Second, in this study, we could not confirm the anesthesia depth in both groups due to lack of facility. Inadequate depth of anesthesia can, to some extent, increase the hemodynamic responses to intubation. 

## 5. Conclusion

Prophylactic dexmedetomidine can attenuate the hemodynamic responses to laryngoscopy and DLT intubation with minimal adverse effects. This application may be useful to patients who are at risk of cardiovascular complications related to severe hypertension and tachycardia after DLT intubation.

## Figures and Tables

**Table 1:** Patient characteristics and operative data.

Variables	Dexmedetomidine group (*n* = 30)	Control group (*n* = 30)
Age (yr)	48.0 ± 19.4	47.8 ± 15.1
Sex		
Female	12 (40)	17 (57)
Male	18 (60)	13 (43)
ASA		
I	11 (37)	8 (27)
II-III	19 (63)	22 (73)
Body mass index (kg/m^2^)	21.2 ± 3.3	20.8 ± 3.3
Concurrent disease		
Hypertension	10 (33)	6 (20)
Diabetes mellitus	3 (10)	1 (3)
Duration of anesthesia (minutes)	172 ± 80	154 ± 68
Duration of surgery (minutes)	142.3 ± 80.4	121.3 ± 67.5
Type of operation		
Thoracoscopy	11 (37)	18 (60)
Thoracotomy	19 (63)	12 (40)
Extent of surgery		
Explorative thoracotomy	9 (31)	11 (39)
Wedge resection	5 (17)	6 (21)
Lobectomy	15 (52)	10 (36)
Pneumonectomy	0	1 (4)
Baseline hemodynamic variables		
SBP (mmHg)	141.6 ± 19.7	136.1 ± 18.9
DBP (mmHg)	78.9 ± 9.6	74.8 ± 12.4
MAP (mmHg)	101.9 ± 12.2	95.9 ± 14.9
HR (beats per minute)	78.4 ± 17.1	81.2 ± 16.7
RPP (mmHg min^−1^)	11,094.0 ± 2,571.2	10,931.7 ± 2,381.8
Intubation time (seconds)	19.8 ± 6.9	20.7 ± 8.7

Data are presented as mean ± SD or number (%).

ASA: The American Society of Anesthesiologists, SBP: systolic blood pressure, DBP: diastolic blood pressure, MAP: mean arterial pressure, HR: heart rate, RPP: rate pressure product.

**Table 2:** The multivariate mean difference of systolic blood pressure, diastolic blood pressure, mean arterial pressure, and heart rate by treatment group adjusted for multiple variables^†^.

	Difference*	*P* value	95% CI
Systolic blood pressure (mmHg)	15.2	<0.001	6.9–23.6
Diastolic blood pressure (mmHg)	10.5	<0.001	5.6–15.3
Mean arterial pressure (mmHg)	14.0	<0.001	7.4–20.6
Heart rate (beats per minute)	10.5	<0.001	6.2–14.8
Rate pressure product (mmHg min^−1^)	2,462.8	<0.001	1,415.7–3,510.0

The mean difference of each hemodynamic variable was estimated from hemodynamic change between baseline, before intubation, during intubation, and T1–T10.

^†^Adjusted for age, gender, ASA classification, preexisting hypertension, baseline hemodynamic variables, intubation time, and intubation attempts.

*Placebo group-treatment group.

**Table:** (a)

Time	SBP (mmHg)			DBP (mmHg)			MAP (mmHg)		
	Dex group (*n* = 30)	Control group (*n* = 30)	*P* value	Dex group (*n* = 30)	Control group (*n* = 30)	*P* value	Dex group (*n* = 30)	Control group (*n* = 30)	*P* value
Baseline	141.6 ± 19.7	135.4 ± 17.6	0.204	79.0 ± 9.6	74.8 ± 12.4	0.151	102 ± 12.2	95.9 ± 14.9	0.092
Before intubation	106.5 ± 20.7^#^	101.9 ± 17.2^#^	0.360	59.2 ± 10.9^#^	59.2 ± 11.1^#^	0.991	75.5 ± 14.9^#^	75.2 ± 13.1^#^	0.934
T0	122.8 ± 31.3^#^	149.4 ± 37.3^#^	0.004*	73.3 ± 23.4	87.4 ± 21.8^#^	0.009*	91.9 ± 28.1^#^	111.2 ± 29.6^#^	0.012*
T1	137.2 ± 36.1	153.4 ± 31.3^#^	0.068	80.7 ± 19.3	88.9 ± 18.5^#^	0.099	101.3 ± 26.6	114.4 ± 26.1^#^	0.058
T2	124.1 ± 30.9^#^	143.9 ± 28.5^#^	0.012*	70.9 ± 15.3^#^	82.2 ± 14.9^#^	0.005*	90.8 ± 22.6^#^	107.1 ± 21.9^#^	0.006*
T3	111.8 ± 22.8^#^	127.3 ± 21.0^#^	0.008*	63.9 ± 12.5^#^	74.2 ± 11.4	0.002*	81.3 ± 16.1^#^	95.2 ± 16.6	<0.001*
T4	104.4 ± 22.8^#^	116.0 ± 16.6^#^	0.013*	60.5 ± 10.1^#^	68.4 ± 10.4^#^	0.004*	76.5 ± 14.0^#^	86.9 ± 12.6^#^	0.002*
T5	102.7 ± 23.0^#^	111.3 ± 16.5^#^	0.017*	59.7 ± 12.0^#^	66.6 ± 11.2^#^	0.025*	75.3 ± 17.0^#^	84.1 ± 12.8^#^	0.008*
T6	99.2 ± 17.6^#^	109.1 ± 16.7^#^	0.028*	57.9 ± 9.6^#^	65.8 ± 11.5^#^	0.006*	72.5 ± 12.8^#^	82.4 ± 13.7^#^	0.005*
T7	97.2 ± 13.2^#^	107.1 ± 16.4^#^	0.012*	56.9 ± 7.7^#^	65.0 ± 11.8^#^	0.003*	71.4 ± 10.4^#^	81.1 ± 13.9^#^	0.003*
T8	97.7 ± 12.9^#^	109.4 ± 17.0^#^	0.003*	57.0 ± 7.2^#^	65.8 ± 11.7^#^	<0.001*	71.5 ± 10.0^#^	82.2 ± 13.9^#^	0.001*
T9	99.4 ± 15.6^#^	109.1 ± 19.1^#^	0.035*	57.9 ± 9.7^#^	66.6 ± 12.9^#^	0.005*	71.8 ± 13.3^#^	81.1 ± 13.9^#^	0.003*
T10	101.1 ± 17.0^#^	112.2 ± 20.9^#^	0.028*	58.8 ± 9.8^#^	67.9 ± 13.5^#^	0.004*	74.1 ± 10.0^#^	84.7 ± 16.3^#^	0.008*

**Table:** (b)

Time	HR (beat per minute)			RPP (mmHg min^−1^)		
	Dex group (*n* = 30)	Control group (*n* = 30)	*P* value	Dex group (*n* = 30)	Control group (*n* = 30)	*P* value
Baseline	78.4 ± 17.1	81.2 ± 16.7	0.529	11,094.1 ± 502.3	10,9317 ± 434.8	0.808
Before intubation	67.3 ± 13.1^#^	78.9 ± 15.1	0.002*	7,174.6 ± 2,050.3^#^	8, 034.5 ± 2,001.2^#^	0.106
T0	79.9 ± 16.7	97.5 ± 14.6^#^	<0.001*	10,011.1 ± 4,242.7	14,855.0 ± 5,259.0^#^	<0.001*
T1	83.3 ± 13.4	96.8 ± 13.7^#^	<0.001*	11,612.6 ± 4,342.8	15,246.7 ± 4,372.6^#^	0.003*
T2	77.8 ± 13.1	93.1 ± 14.5^#^	0.001*	9,791.1 ± 3,678.7^#^	13,682.6 ± 3,708.2^#^	<0.001*
T3	75.8 ± 13.2	89.8 ± 15.5^#^	<0.001*	8,561.6 ± 2,758.6^#^	11,571.8 ± 2,940.7	<0.001*
T4	75.2 ± 14.6	86.4 ± 14.9^#^	0.004*	7,963.7 ± 2,751.3^#^	10,091.2 ± 2,253.7	0.002*
T5	74.3 ± 15.4	85.6 ± 15.5	0.007*	7,798.3 ± 3,228.5^#^	9,530.6 ± 2,313.2^#^	0.020*
T6	72.7 ± 14.4^#^	84.1 ± 15.6	0.005*	7,291.7 ± 2,353.6^#^	9,232.4 ± 2,290.3^#^	0.002*
T7	72.6 ± 14.1^#^	83.4 ± 16.0	0.007*	7,109.1 ± 1,960.8^#^	8,992.1 ± 2,239.0^#^	0.001*
T8	72.6 ± 14.2^#^	82.0 ± 15.9	0.018*	7,131.9 ± 1,865.2^#^	8,979.1 ± 2,207.4^#^	<0.001*
T9	72.6 ± 14.3^#^	83.2 ± 14.8	0.007*	7,260.9 ± 2,005.5^#^	9,195.8 ± 2,449.2^#^	0.001*
T10	72.6 ± 14.1^#^	82.3 ± 14.5	0.011*	7,395.6 ± 2,107.0^#^	9,292.2 ± 2,422.4^#^	0.002*

Data are presented as mean ± SD. Dex group: dexmedetomidine group. SBP: systolic blood pressure. DBP: diastolic blood pressure. MAP: mean arterial pressure. HR: heart rate. RPP: rate pressure product. Baseline refers to the point of time before the study drug is administered. Before intubation refers to the point of time after anesthetic induction just before intubation. T0 is the period during intubation. T1–10 refer to minutes after intubation. *P value < 0.05 compared between groups. ^#^ *P* value < 0.05 compared to baseline values.

**Table 4:** Incidence of adverse effects during and after intubation.

Events	Dexmedetomidine group (*n* = 30)	Control group (*n* = 30)	*P* value
Hypotension			
SBP < 90 mmHg	4 (13%)	1 (3%)	0.313
Hypertension			
SBP > 200 mmHg	1 (3%)	4 (13%)	0.171
Bradycardia			
(HR < 50 beats per minute)	2 (7%)	0	0.496

Data are presented as number (%). SBP: systolic blood pressure, HR: heart rate.

## References

[1] Chhangani SV (2002). Ventilation techniques for 1-lung anesthesia. *Seminars in Anesthesia Perioperative Medicine and Pain*.

[2] Thompson JP, West KJ, Hill AJ (1997). The cardiovascular responses to double lumen endobronchial intubation and the effect of esmolol. *Anaesthesia*.

[3] Fox EJ, Sklar GS, Hill CH, Villanueva R, King BD (1977). Complications related to the pressor response to endotracheal intubation. *Anesthesiology*.

[4] Roy WL, Edelist G, Gilbert B (1979). Myocardial ischemia during non-cardiac surgical procedures in patients with coronary-artery disease. *Anesthesiology*.

[5] Bloor BC, Ward DS, Belleville JP, Maze M (1992). Effects of intravenous dexmedetomidine in humans: II. Hemodynamic changes. *Anesthesiology*.

[6] Khan ZP, Ferguson CN, Jones RM (1999). Alpha-2 and imidazoline receptor agonists. Their pharmacology and therapeutic role. *Anaesthesia*.

[7] Arcangeli A, D’Alò C, Gaspari R (2009). Dexmedetomidine use in general anaesthesia. *Current Drug Targets*.

[8] Basar H, Akpinar S, Doganci N (2008). The effects of preanesthetic, single-dose dexmedetomidine on induction, hemodynamic, and cardiovascular parameters. *Journal of Clinical Anesthesia*.

[9] Dogru K, Arik T, Yildiz K, Bicer C, Madenoglu H, Boyaci A (2007). The effectiveness of intramuscular dexmedetomidine on hemodynamic responses during tracheal intubation and anesthesia induction of hypertensive patients: a randomized, double-blind, placebo-controlled study. *Current Therapeutic Research*.

[10] Kunisawa T, Nagata O, Nagashima M (2009). Dexmedetomidine suppresses the decrease in blood pressure during anesthetic induction and blunts the cardiovascular response to tracheal intubation. *Journal of Clinical Anesthesia*.

[11] Menda F, Köner Ö, Sayin M, Türe H, Imer P, Aykaç B (2010). Dexmedetomidine as an adjunct to anesthetic induction to attenuate hemodynamic response to endotracheal intubation in patients undergoing fast-track CABG. *Annals of Cardiac Anaesthesia*.

[12] Bajwa SJ, Kaur J, Singh A, Gupta S, Sharma V, Panda A (2012). Attenuation of pressor response and dose sparing of opioids and anaesthetics with pre-operative dexmedetomidine. *Indian Journal of Anaesthesia*.

[13] Slinger PD, Campos JH, Miller RD, Eriksson LI, Fleisher LA, Wiener-Kronish JP, Young W (2010). Anesthesia for thoracic surgery. *Miller 's Anesthesia*.

[14] Sugiura S, Seki S, Hidaka K, Masuoka M, Tsuchida H (2007). The hemodynamic effects of landiolol, an ultra-short-acting *β*1-selective blocker, on endotracheal intubation in patients with and without hypertension. *Anesthesia & Analgesia*.

[15] Maguire A, Thompson JP, Guest C, Sadler PJ, Strupish JW, West KJ (2001). Comparison of the effects of intravenous alfentanil and esmolol on the cardiovascular response to double-lumen endobronchial intubation. *Anaesthesia*.

[16] Avidan MS, Zhang L, Burnside BA (2008). Anesthesia awareness and the bispectral index. *The New England Journal of Medicine*.

[17] Robinson BF (1967). Relation of heart rate and systolic blood pressure to the onset of pain in angina pectoris. *Circulation*.

[18] Cokkinos DV, Voridis EM (1976). Constancy of pressure rate product in pacing induced angina pectoris. *British Heart Journal*.

[19] Tobias JD (2007). Dexmedetomidine: applications in pediatric critical care and pediatric anesthesiology. *Pediatric Critical Care Medicine*.

[20] Kovac AL (1996). Controlling the hemodynamic response to laryngoscopy and endotracheal intubation. *Journal of Clinical Anesthesia*.

[21] Lawrence CJ, De Lange S (1997). Effects of a single pre-operative dexmedetomidine dose on isoflurane requirements and peri-operative haemodynamic stability. *Anaesthesia*.

[22] Sulaiman S, Karthekeyan RB, Vakamudi M, Sundar AS, Ravullapalli H, Gandham R (2012). The effects of dexmedetomidine on attenuation of stress response to endotracheal intubation in patients undergoing elective off-pump coronary artery bypass grafting. *Annals of Cardiac Anaesthesia*.

